# Research on a Degradation Identification Method for GIS UHF Partial Discharge Sensors Based on S-Parameters

**DOI:** 10.3390/s25226860

**Published:** 2025-11-10

**Authors:** Tienan Cao, Yufei Cui, Haotian Tan, Wei Lu, Fuzeng Zhang, Kai Liu, Xiaoguo Chen, Lujia Wang

**Affiliations:** 1 Southern Power Grid Scientific Research Institute Co., Ltd., Guangzhou 510700, China; caotn@csg.cn (T.C.); cuiyf@csg.cn (Y.C.); tanht@csg.cn (H.T.); luwei4@csg.cn (W.L.); zhangfz@csg.cn (F.Z.); liukai3@csg.cn (K.L.); chenxg@csg.cn (X.C.); 2 School of Electrical Engineering, China University of Mining and Technology, Xuzhou 221116, China

**Keywords:** GIS, partial discharge, UHF sensor, verification, *S*-parameters

## Abstract

The ultra-high-frequency (UHF) detection method is highly accurate and has a fault localization function. At present, most gas-insulated switchgear (GIS) installations are equipped with online UHF monitoring devices to detect partial discharges. In order to ensure the accuracy of the detection results, UHF sensors need to be verified regularly. UHF sensors used for online monitoring are usually installed at the handhole of the GIS and cannot be removed. Measuring the laboratory verification indexes (e.g., equivalent height, dynamic range, etc.) of the sensors directly is very difficult. However, it is easier to measure *S*_11_ of the sensor for verification and *S*_21_ between it and the neighboring sensors by injecting power signals. Accordingly, this paper proposes a degradation identification method for GIS UHF sensors using a cross-comparison of *S*-parameters. When sensor sensitivity decreases, *S*_11_ increases while *S*_21_ decreases, both serving as effective indicators of performance degradation. In this study, the equivalent *S*-parameter network and the variation mechanisms of *S*_11_ and *S*_21_ during sensor verification were first analyzed. Normal and typically degraded sensor models were then constructed and coupled in different GIS structures for electromagnetic simulation. The simulation and on-site verification results show that *S*_11_ is mainly affected by the sensor’s intrinsic performance and installation conditions at the inspection port, whereas *S*_21_ is predominantly influenced by sensor performance and the propagation characteristics of the GIS structure. Through cross-comparison of *S*_11_ and *S*_21_ at corresponding positions across three phases, sensor aging or failure can be effectively identified, enabling rapid on-site verification without removing the sensors. The proposed method was successfully validated on actual GIS equipment at the China Southern Power Grid Research Institute. It exhibits high accuracy, efficiency, and strong engineering applicability, enabling the early detection of degraded sensors and providing valuable support for condition assessment and maintenance decision-making in GIS online monitoring systems.

## 1. Introduction

In recent years, most gas-insulated switchgear (GIS) installations have been equipped with ultra-high-frequency (UHF) online monitoring systems for the detection of partial discharge (PD), enabling the prompt identification of potential insulation defects [1,2,3]. Some utilities like China Southern Power Grid Company Limited (CSG) will use partial discharge signals to trigger early warnings for protection, sound–light alarms, and other logical features of action in future development. Under the dual requirements of higher power supply reliability and enhanced system safety, early-warning schemes that employ partial discharge (PD) signals as the trigger logic—such as “PD + protection” and “PD + acoustic-optical alarm”—represent an inevitable trend for future development. The above tendency places higher demands on partial discharge UHF detection, especially on the accuracy and reliability of the UHF sensor, which is the main piece of equipment of the detection device [4,5,6]. For this reason, it is imperative to develop a new and effective sensitivity identification procedure for GIS UHF sensors [7,8].

In the early days, since there was no demand for online partial discharge monitoring, the main direction of research was offline UHF sensor verification [9,10]. So-called offline verification is carried out to remove the sensor from the GIS or other electrical equipment, and then it is incorporated into special laboratory verification equipment, which has more calibratable indicators [11,12]. Initially, off-line verification was used to test the effective height of the transducer using a TEM transmission line [13]. Subsequently, coaxial chambers were also used as a test environment for the sweep verification of UHF transducers. Currently, more applications are based on the use of GTEM chambers to calibrate the effective height of UHF transducers and the sensitivity and dynamic range of UHF detection systems. This laboratory verification program is now widely used for UHF detection devices loaded into GIS for the first time [14,15]. However, the sensors used for online monitoring are usually built-in and fixed at the handholes inside the GIS, which makes them difficult to dismantle. As a result, offline calibration often introduces significant errors, since it fails to account for the complex structural characteristics of GIS and the actual operating conditions of sensors within the handhole [16,17]. Under the new requirements for the online monitoring of local discharge signals and protection action, it is imperative to adopt more accurate and faster on-site verification methods for UHF sensors [18,19,20].

Currently, a major predicament in GIS partial discharge sensor monitoring verification is the scarcity of on-site verification indicators [21]. Therefore, there is an urgent need to enhance the on-site verification indicators of GIS UHF sensors to improve accuracy [22,23]. The CIGRE TF15/33.03.05 working group recommended an equivalent 5pC verification method, which was written into the IEC guidelines. Some scholars connected an extended cavity externally on the GIS and pressurized the cavity to induce discharge and verify the monitoring system [24,25]; there is also related research that has been conducted to quantitatively verify disk sensors, ring sensors, and media window sensors of GIS through the correspondence between UHF signals and discharges [26,27]. Universities and scholars have developed various kinds of high-performance UHF sensors and have analyzed the effect of noise interference on local discharge detection [28] and the influence of GIS structure on the propagation characteristics of UHF electromagnetic waves [29]. On this basis, two types of on-site verification programs have been proposed, with certain applications. One is the artificial pulse injection verification method based on the equivalent field strength, which injects the equivalent pulse simulating the local discharge signal into the GIS and verifies the sensitivity of the sensor according to its output response [30]. The other is to measure the *S*_21_ parameter between two sensors and verify the UHF sensor according to the change in *S*_21_. *S*_21_ is the frequency-domain transfer function (frequency response curve) between the two UHF sensors of the GIS. However, just relying on *S*_21_ alone is not sufficient for verifying UHF sensors, especially in complex field working conditions. *S*_11_ is an inherent parameter that characterizes the coupling properties of UHF sensors within the handhole. It represents the fundamental cause of sensor performance variations and exhibits high sensitivity, thereby making it a suitable evaluation metric for on-site calibration. The *S*_11_ parameter can be measured simultaneously with *S*_21_ without adding any extra workload. The GIS ultra-high-frequency (UHF) sensor calibration method based on the comparative analysis of *S*_11_ and *S*_21_ is conducted by additionally measuring the *S*_11_ parameter and subsequently comparing the mean values of *S*_11_ and *S*_21_ for sensors located at corresponding positions in different phases within the detection frequency band. This approach effectively reveals sensor defects that cannot be identified through *S*_21_ measurements alone, thereby enhancing both the accuracy and sensitivity of the calibration results.

In this paper, we first studied the theory for the *S*_11_ parameter of a UHF sensor and the *S*_21_ parameter between UHF sensors in GIS and proposed a verification scheme based on a cross-comparison of *S*_11_ and *S*_21_. Then, the UHF sensor model and the typically degraded UHF sensor model were established through simulation, and the change rule of the UHF sensor’s *S*_11_ and its adjacent sensor’s *S*_21_ in the GIS was verified when the sensitivity of the UHF sensor was degraded. Finally, validation was completed on the GIS in the 500 kV substation of State Grid Ningxia Power Co. Ltd., Ningxia, China. proving the validity and feasibility of the joint cross-comparison of *S*_11_ and *S*_21_.

## 2. Material and Methods

### 2.1. S-Parameter Network

**Figure 1:**
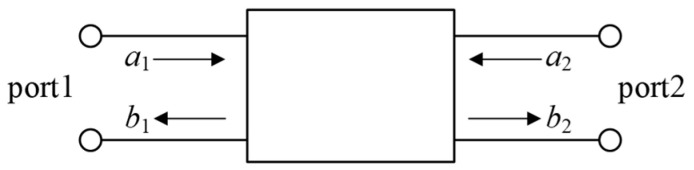
*S*-parameters network.

As shown in Figure 1, the *S*-parameter network is a network of scattering parameters defined at each port connected to a matched load to describe the frequency-domain characteristics of a transmission channel. The *S*-parameters comprise *S*_11_ (i.e., the reflection parameter), *S*_22_ (i.e., the single-ended loss of the output port), *S*_21_ (i.e., the double-ended insertion loss), and *S*_12_ (i.e., the double-ended insertion loss).

According to the electromagnetic wave theory, we have

(1) $$\left[\begin{array}{l} b_{1}\\ b_{2} \end{array}\right]=\left[\begin{array}{l} S_{11}\\ S_{21} \end{array}\right.\left.\begin{array}{l} S_{12}\\ S_{22} \end{array}\right]\left[\begin{array}{l} a_{1}\\ a_{2} \end{array}\right]$$

In Equation (1), *a*_1_ is the refraction of the normalized incident wave; *a*_2_ is the reflection of the normalized incident wave; *b*_1_ is the reflection of the normalized transmitted wave; and *b*_2_ is the refraction of the normalized transmitted wave.

When port 2 is connected to the matched load, *a*_2_ is 0. Equation (1) can be transformed into

(2) $$\left\{\begin{array}{c} S_{11}=\frac{b_{1}}{a_{1}}\\ S_{21}=\frac{b_{2}}{a_{1}} \end{array}\right.$$

**Figure 2:**
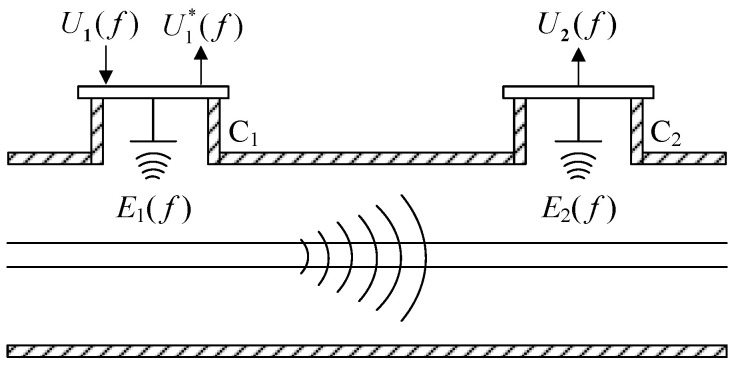
Wave process during GIS verification.

UHF sensors used for online monitoring are often coupled inside the handhole in a built-in manner. Taking sensors C_1_ and C_2_ as an example, the wave process during on-site verification of UHF sensors in GIS is shown in Figure 2. Antennas are reciprocal, i.e., the same antenna can either inject power signals to excite UHF electromagnetic waves or inject UHF electromagnetic waves to couple power signals. Therefore, sensor C_1_ may be used as a transmitting sensor and sensor C_2_, adjacent to C_1_, may be used as a receiving sensor for verification.

After injecting the power signal *U*_1_(*f*) into C_1_, C_1_ will excite a UHF electromagnetic wave *E*_1_(*f*). Part of *E*_1_(*f*) forms a standing wave at the handhole, and the other part of *E*_1_(*f*) is a traveling wave propagating in the GIS cavity. Sensor C_1_ receives the standing wave of its own excitation and outputs the reflected signal $U_{1}^{*}(f)$. The traveling wave propagates in the GIS cavity with many refractions, reflections, and superposition and reaches the receiving sensor C_2_ when it has been attenuated into *E*_2_(*f*). C_2_ receives the attenuated electromagnetic wave *E*_2_(*f*) and then outputs the voltage *U*_2_(*f*).

The above wave process can be equated to the *S*-parameter network of Figure 1. In Figure 2, the feed terminal of the injecting sensor C_1_ is equivalent to port 1 in Figure 1; *U*_1_(*f*) is considered a1; $U_{1}^{*}(f)$ is considered *b*1; the feed terminal of the receiving sensor C_2_ is port 2; and *U*_2_(*f*) is considered *b*_2_. Then, from Equation (2), the defining equations for the real parts of *S*_11_ and *S*_21_ of C_1_ at the time of verification of the GIS UHF sensor are

(3) $$\left\{\begin{array}{c} S_{11}=20\mathrm{lg}\frac{U_{1}^{*}(f)}{U_{1}(f)}\\ S_{21}=20\mathrm{lg}\frac{U_{2}(f)}{U_{1}(f)} \end{array}\right.$$

In Equation (3), *U*_1_(*f*) is the voltage injected into the sensor C_1_; $U_{1}^{*}(f)$ is the voltage reflected from the injecting sensor C_1_; and *U*_2_(*f*) is the voltage output from the receiving sensor C_2_.

### 2.2. S_11_ and S_21_ Change Mechanism

Disregarding the signal leakage and energy loss, the UHF electromagnetic wave incident in the GIS cavity should be equal to the sum of the reflected and radiated phases. Therefore, the *S*_11_ phase quantity representing the reflection characteristics and the *S*_21_ phase quantity representing the radiation characteristics have the following relationship in the frequency domain:

(4) $${\left|S_{11}\right|}^{2}+{\left|S_{21}\right|}^{2}=1$$

The *S*-parameters tested during field verification are similar to the transfer functions in the frequency domain, and they all reflect only the intrinsic properties of the object. *S*_11_ is related only to the performance of the sensor itself and the size of the handhole, determined by the standing waves of the electromagnetic wave, reflecting the operating performance of the UHF sensor after it is mounted in a narrow space within the handhole. *S*_21_ is related only to the characteristics of the sensor itself and the structure of the GIS cavity, determined by the traveling waves of electromagnetic waves, reflecting the performance of the sensor in receiving the UHF signals propagating over the far end of the cavity. Since it is unlikely that the handhole and chamber structure will change during verification, the only reason for the change in *S*_11_ and *S*_21_ during the actual verification is a degradation in sensor performance.

If the performance of the sensor degrades, the amount of reflection will increase and the amount of radiation will decrease when a signal is injected into this sensor. Thus, its *S*_11_ in the free space will increase to varying degrees, which in turn will lead to an increase in *S*_11_ tested during the calibration at the handhole. From Equation (4), it can be seen that its *S*_21_ will decrease at the same time.

The *S*-parameter is a phase quantity written in complex form, while the *S*_11_ and *S*_21_ spectra obtained from the actual verification using the network analyzer are both given in terms of their real parts. Therefore, when only *S*_21_ is tested with the network analyzer, it is possible that the variation in the imaginary part is large while the variation in the real part is not large and thus the results are not obvious, resulting in missed detection. Therefore, it is necessary to measure both the *S*_11_ of a sensor and the *S*_21_ of this sensor and its adjacent sensors and then make a comprehensive judgment.

### 2.3. Verification Method Based on Cross-Comparison of S_11_ and S_21_

The core feature of the joint cross-comparison verification method based on *S*_11_ and *S*_21_ is the three-phase symmetry of the UHF sensors installed on the GIS. Since the A, B, and C phases of the GIS are three-phase-symmetric, if the sensors are normal, the measured *S*_11_ and *S*_21_ curves of three-phase sensors at the corresponding positions are similar, and the calculated mean values are also similar. However, if there is a sensor with degraded performance, according to the theory of change of the S-parameter analyzed above, either *S*_11_ and *S*_21_ of this sensor, or both, will change significantly compared to the corresponding position sensors of the other two phases.

**Figure 3:**
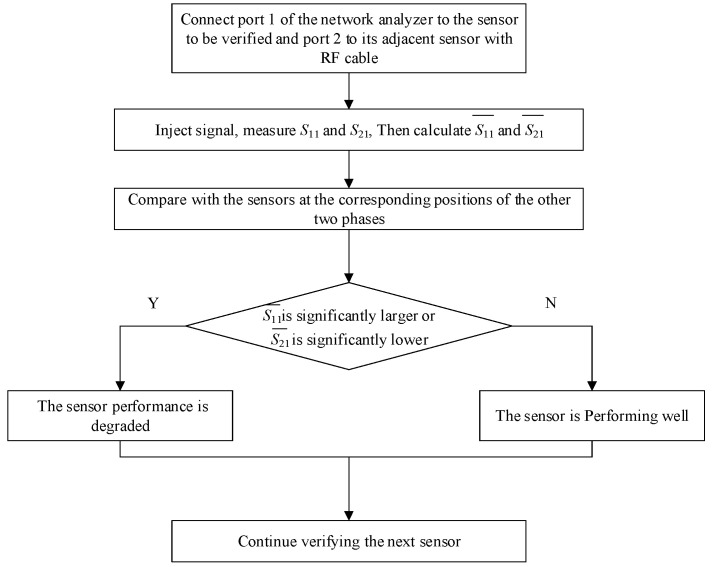
UHF sensor verification process based on S_11_ and S_21_ cross-comparison.

Prior to the initiation of the measurement procedure, the network analyzer was calibrated to a reference power level of 0 dBm. An RF cable is used to connect the network analyzer, the sensor to be verified, and the sensor adjacent to the sensor to be verified. A power signal is injected into the sensor to be verified, and the *S*_11_ of that sensor and the *S*_21_ between the two sensors are measured. After completing this test phase, the equipment is rewired to measure *S*_11_ and *S*_21_ at the other corresponding positions for each phase, and the mean values of *S*_11_ and *S*_21_ are calculated from Equations (5) and (6). Finally, $\bar{S_{11}}$ and $\bar{S_{21}}$ are compared with each other between phases A, B, and C. A flowchart of this verification method is shown in Figure 3.

(5) $$\bar{S_{11}}=\frac{1}{f_{\max}-f_{\min}}\int_{f_{\min}}^{f_{\max}}S_{11}$$

(6) $$\bar{S_{21}}=\frac{1}{f_{\max}-f_{\min}}\int_{f_{\min}}^{f_{\max}}S_{21}$$

In Equations (5) and (6), *f*_min_ is the lowest frequency point of the frequency sweep during verification and fmax is the highest frequency point of the frequency sweep.

## 3. Simulations

In order to verify the feasibility of the above joint cross-comparison verification based on the *S*_11_ and *S*_21_ parameters, UHF sensor and degradation sensor models were established in CST, the simulated sensors were coupled in built-in form at the handhole of the straight-cavity GIS, and the simulation results of each UHF sensor’s verification in GIS were analyzed by cross-comparison.

### 3.1. Modeling of the UHF Sensor

**Figure 4:**
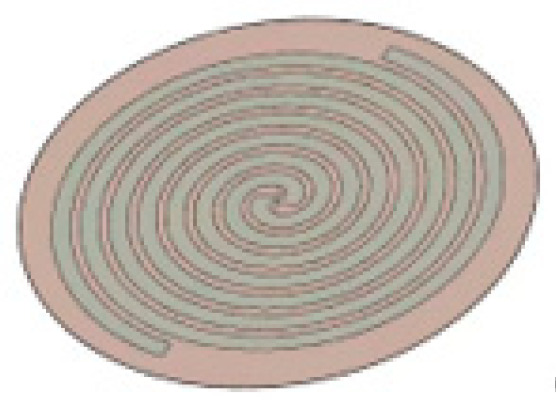
UHF antenna simulation model.

The UHF sensor is an ultra-wideband antenna with a frequency band range of approximately 0.3–1.8 GHz. The antenna receives electromagnetic waves in that band and converts them into voltage signals. In this paper, UHF sensors were modeled with an Archimedes spiral antenna commonly used in engineering, which is small, simple, and has unique advantages in fault location. The established model A planar Archimedean spiral antenna is shown in Figure 4. The material of antenna A’s wire arm was set to be PEC and the substrate was set to be PTFE. The wire arm consists of two Archimedean solenoids whose sagittal length increases linearly with increasing angle. Its mathematical model is

(7) $$\left\{\begin{array}{l} r_{1}=r_{0}+\alpha \varphi \\ r_{2}=r_{1}+\alpha \varphi \end{array}\right.$$

In Equation (7), *r*_0_ is the distance between the helix starting point and the coordinate center, i.e., the inner diameter; *r*_1_ and *r*_2_ are the distances from any point on the line arm to the coordinate center; *α* is the helix rate; and *φ* is the angle at which the helix is turned.

According to the radiation characteristics of the Archimedean spiral antenna, the inner radius *r*_0_ is determined by the highest operating frequency (corresponding to the minimum wavelength), while the outer radius *r*_M_ is determined by the lowest operating frequency (corresponding to the maximum wavelength). The typical selection criteria are as follows:

(8) $$\left\{\begin{array}{l} 2r_{0}<\lambda_{\min}/4\\ 2\pi r_{M}>1.25\lambda_{\max} \end{array}\right.$$

Considering the miniaturization requirements of built-in antennas in GIS, the outer radius of the antenna was reduced on the basis of Equation (8). Both the inner and outer radii influence the impedance characteristics of the antenna as well as the installation of the feed balloon. Therefore, the inner and outer radii were determined through simulation-based tuning and optimization. Furthermore, the linewidth *W*, number of turns *N*, and other parameters were derived according to the relational expressions of the Archimedean spiral antenna’s radiation surface. Based on the parameters listed in Table 1, the model C_1_ UHF sensor was established, with its simulation structure shown in Figure 4.

Extensive testing of ultra-high-frequency (UHF) sensors with significant performance degradation has revealed that the primary causes of deterioration include aging, corrosion, and functional failure. Among these, aging is identified as the predominant factor, which is mainly manifested by changes in the physicochemical properties of the antenna after prolonged operation. The corresponding simulation approach involves increasing the helical structure of the antenna and the relative dielectric constant of the substrate to mimic the deterioration of electrical characteristics caused by thermal effects. In a smaller proportion of sensors, corrosion occurs in the antenna helix, which adversely affects the impedance characteristics and operating frequency band. This phenomenon can be simulated by adjusting the linewidth and number of turns of the helix, thereby representing the loss of functionality due to corrosion. Furthermore, sensor failure leads to insufficient current passing through the feed port, making it difficult to generate adequate electromagnetic waves. Such failures can be equivalently modeled by disconnecting the feed port of the antenna.

Based on the aforementioned simulation methods of degraded ultra-high-frequency (UHF) sensors and the characteristic parameters observed in practically deteriorated devices, sensor A was employed as the reference model. By increasing the relative permittivity, increasing the conductivity, reducing the linewidth of the Archimedean screw, reducing the number of turns, and disconnecting the antenna feed ports, the degraded UHF sensor models B, C, and D were created. The specific parameter modifications for each degraded sensor are summarized in Table 2.

**Figure 5:**
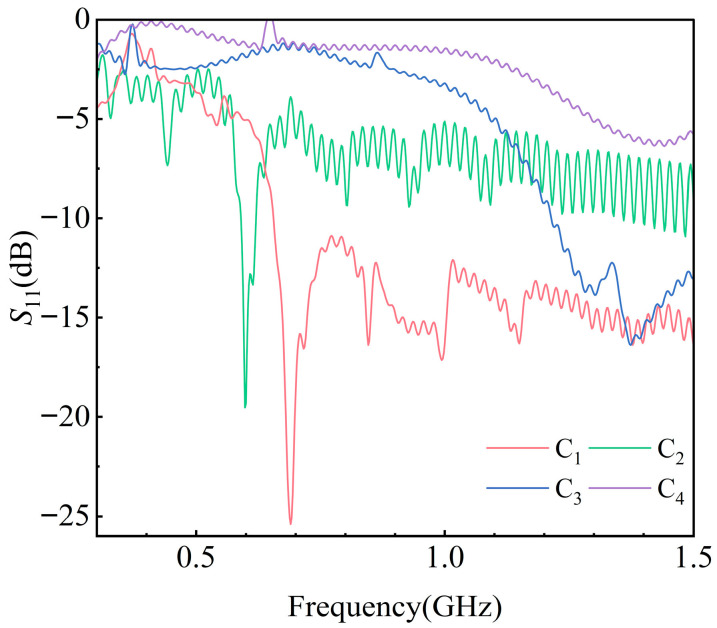
S_11_ of sensors with different performances.

Based on the aforementioned model, the antenna feed port was set as a wave port. Since the energy density is mainly concentrated below 1.5 GHz, the simulation frequency range was defined as 0.3–1.5 GHz. The *S*_11_ curves of antennas C_1_, C_2_, C_3_, and C_4_ simulated in free space are shown in Figure 5.

As illustrated in Figure 5, the *S*_11_ responses exhibit considerable variation across different frequency points, accompanied by noticeable oscillations. This behavior is primarily attributed to the frequency-dependent characteristics of UHF sensors. Each *S*_11_ curve demonstrates a minimum point, which corresponds to the resonant frequency of the sensor.

For antenna C_1_, the *S*_11_ values remain below −10 dB at frequencies above 0.65 GHz, indicating that its performance satisfies engineering application requirements. In contrast, the adjustment of the local relative permittivity of antenna C_2_ prevented the generation of equal but opposite-phase currents, resulting in insufficient excitation of electromagnetic waves and an overall inferior performance compared with C_1_. For antenna C_3_, the reduction in coil turns altered the effective operating frequency band, leading to a significant increase in *S*_11_ within the detection range. For antenna C_4_, the disconnection of the feed port caused a severe decline in feeding performance, drastically reducing the radiated electromagnetic energy across the entire frequency band. The average values of *S*_11_ within the detection band, calculated according to Equation (5), are summarized in Table 3.

As shown in Table 3, the increase in the *S*_11_ curves of the degraded sensors inevitably leads to an increase in their average values. This average value can therefore serve as a characteristic parameter for identifying sensor performance.

### 3.2. GIS UHF Sensor Verification Model

The model simulated the most common straight-cavity structure in 500 kV GIS. In order to reduce the electromagnetic wave leakage, insulated basins were set at both ends of the GIS to close the cavity. Handholes A and B were punched through the outer wall of the GIS and the sensors were coupled at the handholes, with the requirement that the receiving side of the sensors face the GIS cavity. In order to not distort the original electric field of the cavity, the sensor’s receiving surface should not be smaller than the outer wall of the GIS.

**Figure 6:**
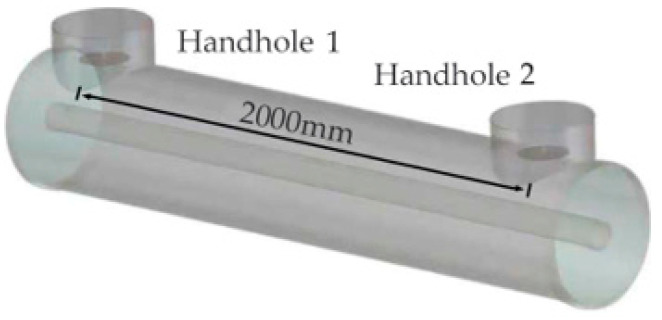
GIS verification model for each UHF sensor.

The inner diameter of the GIS high-voltage conductor was set to 100 mm, the outer diameter was set to 500 mm, the thickness of the outer shell was set to 10 mm, and the distance between both sensors in the GIS was 2000 mm. The material of both the shell and inner conductor was set to PEC, and the relative dielectric constant of the tub insulator was set to 4.6. The port was set to a wave port, the simulation frequency was set to 0.3–1.5 GHz, the boundary conditions were set to an open boundary, and time-domain full-wave simulation was used. The simulation model is shown in Figure 6.

Four different GIS UHF sensor verification models can be obtained by always placing the above-modeled UHF sensor C_1_ at handhole 1 and placing C_1_ and the degraded sensors C_2_, C_3_, and C_4_ at handhole 2, respectively. The same power signal was injected into handhole 2, and the *S*_11_ of the sensor at handhole 2 and the *S*_21_ between the two sensors were tested for the four models.

### 3.3. Analysis of Simulation Results

**Figure 7:**
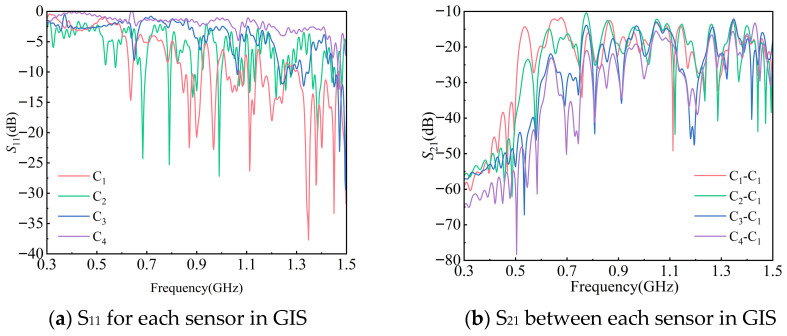
S-parameter curves from simulation.

Due to the different performances of each sensor, the excited traveling waves have different wave processes within the GIS. Each model can be obtained as the *S*_11_ curve for each degraded sensor and the *S*_21_ curve between the normal sensor and each degraded sensor, respectively. Among them, the *S*_11_ for sensors C_1_, C_2_, C_3_, and C_4_ at GIS handhole 2 is shown in Figure 7a. The *S*_21_ between two sensors, C_1_-C_1_, C_2_-C_1_, C_3_-C_1_, and C_4_-C_1_, of the GIS is shown in Figure 7b.

*S*_11_ and *S*_21_ in Figure 6 are calculated as Equations (5) and (6) for the mean values in the detection band range, and the results of the calculations are summarized in Table 4.

The data calculated in Table 4 were analyzed:

(1) When UHF sensors for online monitoring are coupled to the handhole, the handhole is approximately equivalent to a circular waveguide, which has a ‘high-pass’ characteristic for *S*_11_, and the low-frequency band rises significantly when the low-frequency band is repeatedly refracted within the handhole. Compared to free space, the low-frequency band performance decreases when the sensor is operated inside the handhole.

(2) When the performance of a UHF sensor is degraded, the sensor’s ability to effectively radiate the signal is diminished and the reflected signal increases. This sensor’s $\bar{S_{11}}$ in free space rises, which leads to a rise in its $\bar{S_{11}}$ in the handhole. *S*_11_ can be used as an indicator for on-site verification.

When the performance of a UHF sensor is degraded, the $\bar{S_{21}}$ between that sensor and its adjacent sensors is bound to drop due to the limitations of the passive reciprocal *S*-parameter network. *S*_21_ can be used as an indicator for on-site verification.

## 4. Experiments

**Figure 8:**
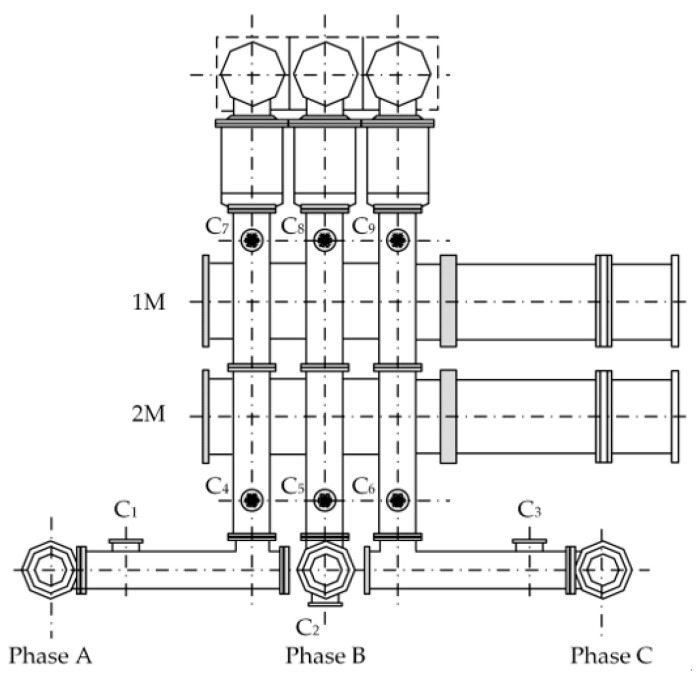
Schematic diagram of the GIS structure for verification.

To verify the correctness of the proposed GIS UHF sensor verification method based on *S*_11_ and *S*_21_, we conducted on-site verification of UHF sensors of 500 kV GIS at a substation of Southern Power Grid Scientific Research Institute Co., Ltd., Guangzhou, China. This GIS structure is shown in Figure 8, containing typical gas chambers such as a straight-axis chamber, an L-shaped chamber, and an insulator structure, and the sensor numbering for this section is also shown in Figure 8. Field verification requires test instruments, including a network analyzer, an RF cable, an N-adapter, and a multimeter. The specific parameters of the test instruments are listed in Table 5.

### 4.1. Verification Process

The verification process is shown in Figure 3. The following is an example of cross-comparison verification for C_1_, C_2_, and C_3_, which illustrates the specific experimental method of UHF sensor verification.

Firstly, the UHF sensor C_1_ is verified in phase A. Port 1 of the network analyzer is connected to the port of sensor C_1_ using an RF cable, and port 2 is connected to the port of sensor C_4_ adjacent to C_1_. To measure the *S*_11_ of sensor C_1_, the *S*_11_ test option is selected in the network analyzer to sweep and save the data. To measure the *S*_21_ between C_1_ and C_4_, the *S*_21_ or *S*_12_ test option is selected in the network analyzer for sweeping and data saving. Next, sensor C_2_ of phase B is verified according to the wiring method described above, and then the *S*_11_ of C_2_ and *S*_21_ of C_2_-C_5_ are measured in turn. Finally, sensor C_3_ of phase C is verified in the same way, and *S*_11_ of C_3_ and *S*_21_ of C_3_-C_6_ are measured in turn. The mean value of the above curves is then calculate after the *S*-parameter curves of the three phases have been measured.

The GIS has a three-phase symmetrical structure, and the positions of the three-phase UHF sensors correspond to each other. For sensors at corresponding positions of different phases, both the $\bar{S_{11}}$ of the sensors and the $\bar{S_{21}}$ between the sensors should be essentially equivalent under normal conditions. Therefore, $\bar{S_{11}}$ should be essentially equal for C_1_, C_2_, and C_3_, and $\bar{S_{21}}$ should be essentially equal for C_1_-C_4_, C_2_-C_5_, and C_3_-C_6_. If the $\bar{S_{11}}$ of a sensor is significantly higher than that of the other sensors or the $\bar{S_{21}}$ between an adjacent sensor is significantly lower than that of the sensor at its corresponding position, it can be judged that the performance of that sensor is degraded.

### 4.2. Analysis of Experimental Results

**Figure 9:**
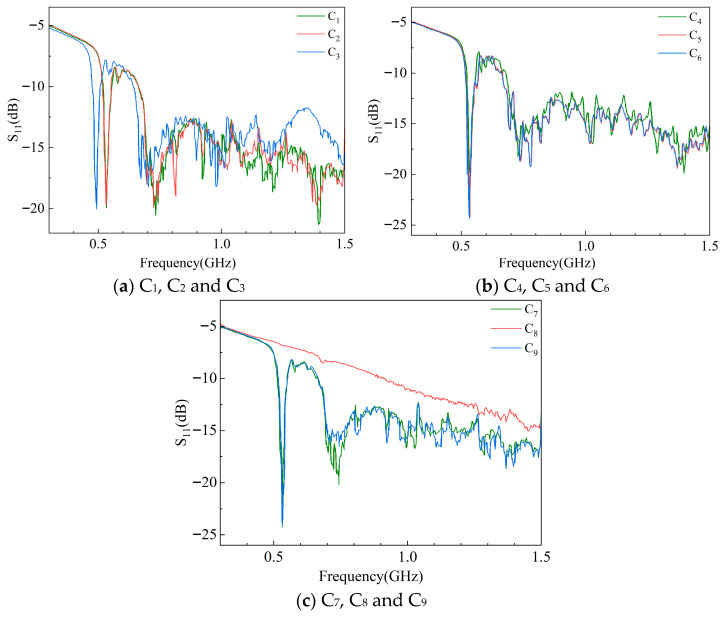
S_11_ curves of sensors measured on-site.

**Figure 10:**
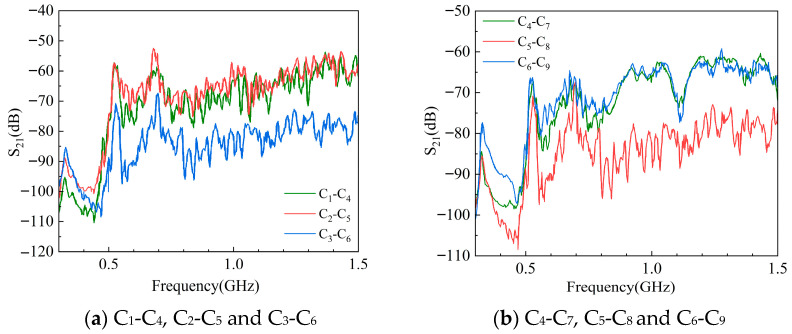
S_21_ curves between sensors measured on-site.

According to the above verification process, the measured *S*_11_ and *S*_21_ curves of the 500 KV GIS UHF sensor are shown in Figure 9 and Figure 10.

The measured *S*_11_ and *S*_21_ are basically similar to the waveforms obtained from simulation, but the curves oscillate more violently due to the large electromagnetic interference in the field. *S*_11_ has poor performance in the low-frequency band and has the lowest resonance point; *S*_21_ is small in the low-frequency band, increases with frequency, reaches its highest point, and then begins to decline and oscillate continuously. The reason for the overall lower *S*_21_ is that the two adjacent sensors of the actual GIS are farther apart and the double-ended insertion loss is greater.

The measured $\bar{S_{11}}$ of each sensor and the $\bar{S_{21}}$ between sensors can be obtained after calculation using Equations (5) and (6). In order to qualitatively describe the variation in $\bar{S_{11}}$ and $\bar{S_{21}}$ during the on-site verification of the GIS UHF sensors, the concept of percentage deviation was introduced. The defining equations for percentage deviation are

(9) $$\sigma_{11}=\frac{\bar{S_{11}}-\bar{S_{b}}}{\bar{S_{b}}}$$

(10) $$\sigma_{21}=\frac{\bar{S_{21}}-\bar{S_{b}}}{\bar{S_{b}}}$$

In Equations (9) and (10), $\bar{S_{b}}$ is the $\bar{S_{11}}$ or $\bar{S_{21}}$ of the reference phase, and the phase in which the sensor has the best verification index at the corresponding positions of the three phases is selected as the reference phase. Therefore, the reference phase of $\sigma_{11}$ is the phase with the smallest $\bar{S_{11}}$ at the corresponding three-phase position, and the reference phase of $\sigma_{21}$ is the phase with the largest $\bar{S_{21}}$ at the corresponding three-phase position.

The results of the above calculations and analyses are summarized in Table 6 and Table 7. The $\bar{S_{11}}$ of sensors C_1_, C_2_, and C_3_, the $\bar{S_{11}}$ of sensors C_4_, C_5_, and C_6_, and the $\bar{S_{11}}$ of sensors C_7_, C_8_, and C_9_ are compared. The $\bar{S_{21}}$ between sensors C_1_-C_4_, C_2_-C_5_, and C_3_-C_6_ and the $\bar{S_{21}}$ between sensors C_4_-C_7_, C_5_-C_8_, and C_6_-C_9_ are also compared.

Since the substation is a strong electromagnetic interference environment, the measured *S*_11_ and *S*_21_ of the three-phase sensors with good performance at their corresponding positions will have a small deviation from each other, which will lead to a minor deviation for the calculated mean value. When the deviation amount of *S*_11_ or the deviation amount of *S*_21_ exceeds 10%, it is considered that the *S*-parameter of this sensor has a larger deviation than the other two phases. This is mainly because a 10% deviation is already much more than the deviation due to random disturbances on-site. Thus, the performance of this sensor may be degraded and needs to be further checked with a multimeter.

The data calculated in Table 6 and Table 7 were analyzed:

(1) The difference between the $\bar{S_{11}}$ of sensors C_1_ and C_2_ was very small, while the $\bar{S_{11}}$ of C_3_ rose by 11.78% compared to C_1_, and the percentage deviation of $\bar{S_{11}}$ was larger, exceeding 10%. The difference between the $\bar{S_{21}}$ of C_1_-C_4_ and C_2_-C_5_ was smaller, while the $\bar{S_{21}}$ of C_3_-C_6_ also decreased by 26.81% compared to the smallest value for C_2_-C_5_ at the corresponding position. As a result, UHF sensor C_3_ may have undergone performance degradation. After disassembly and further inspection, C_3_ was found to have deteriorated due to long-term high-temperature operation.

(2) The $\bar{S_{11}}$ of C_7_ and C_9_ were closer, while the $\bar{S_{11}}$ of C_8_ increased by 26.60% over C_7_. The difference in $\bar{S_{21}}$ between C_4_-C_7_ and C_6_-C_9_ was very small, while the $\bar{S_{21}}$ of C_5_-C_8_ decreased by 20.71% compared to C_6_-C_9_. Compared to the sensors at the corresponding positions in phases A and C, both $\bar{S_{11}}$ and $\bar{S_{21}}$ of C_8_ underwent significant deviations. By using a multimeter to measure the resistance of sensor C_8_, it could be found that its resistance value was close to 0. A short-circuit fault may have occurred inside this sensor.

(3) Apart from sensors C_3_ and C_8_, the other UHF sensors in Figure 8 did not show significant deviations while verifying $\bar{S_{11}}$ and $\bar{S_{21}}$, indicating that these sensors performed well overall and did not undergo significant degradation.

(4) *S*-parameters are phase quantities, and the *S*_11_ and *S*_21_ measured during verification were given as their real parts. If *S*_11_ or *S*_21_ is used alone as a verification index, when the performance of a UHF sensor decreases, there may be a situation in which the real part does not change much, which in turn affects the accuracy of the verification results. In addition, *S*_11_ has better interference immunity compared to *S*_21_. Therefore, the $\bar{S_{11}}$ and $\bar{S_{21}}$ of the sensors corresponding to the structure of the three phases A, B, and C of the GIS should be compared with each other at the same time during verification. When one of the deviation percentages of $\bar{S_{11}}$ or $\bar{S_{21}}$ exceeds 10%, the sensor should be further checked even if the other *S*-parameter indicator does not change too much.

## 5. Conclusions

Although the proposed verification method demonstrated good performance in distinguishing between various types of sensor degradation, the influence of sensor sensitivity on the accuracy of S-parameter assessment was not quantitatively analyzed in this study. The sensitivity of UHF sensors may vary with installation conditions, aging level, and signal coupling efficiency, which could introduce additional uncertainty into the verification results. A detailed sensitivity analysis and quantitative evaluation will therefore be included in our future research work.

The simulation model developed in this study verified the fundamental characteristics of *S*_11_ and *S*_21_ during the calibration of GIS ultra-high-frequency (UHF) sensors. When the performance of the UHF sensor decreases, its ability to radiate an effective signal decreases and the amount of reflection increases, which can result in a rise in *S*_11_ measured at the handhole for that sensor as well as a fall in *S*_21_ between adjacent sensors. When the performance of the sensor is degraded, in some cases *S*_21_ may not change much. The *S*_11_ of the sensor to be verified and its *S*_21_ with the adjacent sensors should be measured at the same time, and a judgment should be made by combining the changes in *S*_11_ and *S*_21_ to improve the accuracy of the verification results.

GIS is a three-phase symmetry. When the sensor is normal, the *S*_11_ and *S*_21_ of the three-phase sensor corresponding to its position should be similar, and the average value of *S*_11_ and *S*_21_ should be close. When the performance of the sensor is degraded, there is an $\bar{S_{11}}$ rise or $\bar{S_{21}}$ fall compared to the other sensors in their corresponding positions. In this paper, the degraded sensors were modeled, and they were coupled in GIS to simulate the real situation of on-site verification, which verified the correctness of the proposed theory of *S*_11_ and *S*_21_ changes. Based on the simulation, the *S*_11_ of the sensor and the *S*_21_ between the sensors were measured on the 500 kV GIS of the Southern Power Grid Scientific Research Institute Co., Ltd., and then analyzed according to the cross-comparison method. The comparative analysis of *S*_11_ and *S*_21_ has been demonstrated to be an effective method for verifying the performance of on-site ultra-high-frequency (UHF) sensors [31,32]. Furthermore, the proposed simulation model exhibits high accuracy and reliability, providing a solid foundation for subsequent studies on the calibration of GIS UHF sensors.

## Figures and Tables

**Table 1:** Parameter setting for sensor C_1_.

Parameter	Assigned Value
Spiral inner diameter 2 *r*_0_/mm	10
Spiral outer diameter *r*_m_/mm	166
Spiral linewidth *W*/mm	7.7
Number of turns *N*	3.7
Spiral thickness *D*/mm	0.035
Substrate radius *r*/mm	90
Substrate thickness *d*/mm	0.15
Substrate relative permittivity *ε*_r_	4.6

**Table 2:** Parameter adjustment for each typical deteriorated sensor.

Sensor ID	Parameter Adjustment
C_2_	Local relative permittivity *ε*r increased to 6
C_3_	coil turns; N reduced to 1.7
C_4_	Antenna feed port disconnected

**Table 3:** $\bar{S_{11}}$ for each sensor model.

Calibration Index	C_1_	C_2_	C_3_	C_4_
$\bar{S_{11}}$	−11.343	−6.607	−5.459	−2.315

**Table 4:** Data analysis for each sensor.

Sensor Number	$\bar{S_{11}}$/dB	$\bar{S_{21}}$/dB
C_1_	−9.264	−24.513
C_2_	−6.382	−25.681
C_3_	−4.610	−29.520
C_4_	−2.052	−33.441

**Table 5:** UHF verification equipment and its parameters.

Serial	Name	Performance Parameters
1	multimeter	The maximum rated voltage should be no less than 800 V AC
2	N-type adapter	L-shaped 2 pieces (male connects to female), T-shaped 2 pieces (1 male connector and 2 female connectors)
3	RF cable	The attenuation characteristic should not exceed 3 dB
4	network analyzer	S_11_, S_21_ parametric test function frequency range: 1 MHz to 6.5 GHz test frequency interval not greater than 2 MHz
5	UHF sensor	frequency range: 500 MHz to 1500 MHz sensitivity: −70 dBm gain: 20 dBm

**Table 6:** $\bar{S_{11}}$ analysis for each sensor.

Sensor Number	$\bar{S_{11}}$/dB	$\sigma_{11}$/%	Qualified
C_1_	−15.309	0	yes
C_2_	−14.471	5.47	yes
C_3_	−13.505	11.78	no
C_4_	−13.755	4.91	yes
C_5_	−14.335	0.90	yes
C_6_	−14.465	0	yes
C_7_	−14.077	1.08	yes
C_8_	−10.445	26.60	no
C_9_	−14.231	0	yes

**Table 7:** $\bar{S_{21}}$ analysis between each sensor.

Sensor Number	$\bar{S_{11}}$/dB	$\sigma_{21}$/%	Qualified
C_1_-C_4_	−72.129	7.72	yes
C_2_-C_5_	−66.959	0	yes
C_3_-C_6_	−84.915	26.81	no
C_4_-C_7_	−73.217	3.96	yes
C_5_-C_8_	−85.018	20.71	no
C_6_-C_9_	−70.430	0	yes

## Data Availability

Data are contained within the article.
